# Smooth and flat phase-locked Kerr frequency comb generation by higher order mode suppression

**DOI:** 10.1038/srep26255

**Published:** 2016-05-16

**Authors:** S.-W. Huang, H. Liu, J. Yang, M. Yu, D.-L. Kwong, C. W. Wong

**Affiliations:** 1 Mesoscopic Optics and Quantum Electronics Laboratory, University of California Los Angeles, CA, USA; 2 Institute of Microelectronics, Singapore, Singapore

## Abstract

High-*Q* microresonator is perceived as a promising platform for optical frequency comb generation, via dissipative soliton formation. In order to achieve a higher quality factor and obtain the necessary anomalous dispersion, multi-mode waveguides were previously implemented in Si_3_N_4_ microresonators. However, coupling between different transverse mode families in multi-mode waveguides results in periodic disruption of dispersion and quality factor, and consequently causes perturbation to dissipative soliton formation and amplitude modulation to the corresponding spectrum. Careful choice of pump wavelength to avoid the mode crossing region is thus critical in conventional Si_3_N_4_ microresonators. Here, we report a novel design of Si_3_N_4_ microresonator in which single-mode operation, high quality factor, and anomalous dispersion are attained simultaneously. The novel microresonator is consisted of uniform single-mode waveguides in the semi-circle region, to eliminate bending induced mode coupling, and adiabatically tapered waveguides in the straight region, to avoid excitation of higher order modes. The intrinsic quality factor of the microresonator reaches 1.36 × 10^6^ while the group velocity dispersion remains to be anomalous at −50 fs^2^/mm. With this novel microresonator, we demonstrate that broadband phase-locked Kerr frequency combs with flat and smooth spectra can be generated by pumping at any resonances in the optical C-band.

Optical frequency combs, unique light sources that coherently link optical frequencies with microwave electrical signals, have made a broad impact on frequency metrology, optical clockwork, precision navigation, and high speed communication over the past decades^1^,^2^. Parametric oscillation in ultrahigh *Q* microresonators^3^,^4^, facilitated by the high quality factors and the small mode volumes, is an alternative physical process that offers the opportunity of optical frequency comb generation in compact footprints^5^. The recent demonstrations of octave spanning parametric oscillation^6^,^7^, low-phase noise photonic oscillator^8^,^9^,^10^,^11^, stabilized optical frequency microcomb^12^,^13^,^14^, and mode-locked femtosecond pulse train^15^,^16^,^17^,^18^,^19^ have triggered great excitements. In particular, the observation of dissipative soliton formation and soliton-induced Cherenkov radiation^20^ offers a reliable route towards self-referenced broadband optical frequency microcomb. Dissipative solitons are localized attractors where the Kerr nonlinearity is compensated by the cavity dispersion and the cavity loss is balanced by the parametric gain^21^. Thus the cavity dispersion and the pump-resonance detuning are two determining parameters in the existence of dissipative solitons in ultrahigh *Q* microresonators.

Generation of microresonator-based optical frequency comb, or Kerr frequency comb, has been studied in various material platforms^10^,^22^,^23^,^24^,^25^, including Si_3_N_4_ planar waveguide system that is especially suitable for monolithic electronic and feedback integration. For Si_3_N_4_ microresonators, dispersion is typically engineered by the design of waveguide geometry. Anomalous dispersion, required for bright dissipative soliton formation, is achieved using multi-mode waveguides in the optical C/L-band wavelength range. Besides, scattering loss in multi-mode waveguides is reduced, leading to higher quality factors and lower comb generation threshold^26^. However, coupling between different transverse mode families in the multi-mode waveguides results in periodic disruption of dispersion and quality factor, introducing additional perturbation to the dynamics of Kerr frequency comb and dissipative soliton generation^27^,^28^,^29^,^30^,^31^. Such effect manifests itself as characteristic amplitude modulation in the Kerr frequency comb spectrum or detrimental destabilization of the dissipative cavity soliton, depending on the strength and position of the mode coupling^29^,^31^. Careful choice of pump mode to avoid the mode crossing region and increase of cavity’s free spectral range (FSR) are thus necessary for dissipative soliton formation in conventional Si_3_N_4_ microresonators.

Here we report a novel design of Si_3_N_4_ microresonator in which single-mode operation, high quality factor, and anomalous dispersion are attained simultaneously. No higher order mode is observed throughout the optical C/L-band in the transmission spectrum. A high resolution coherent swept wavelength interferometer is implemented to determine the intrinsic quality factor and the group velocity dispersion (GVD, *β*_*2*_) of the microresonator. They are measured at 1.36 × 10^6^ and −50 fs^2^/mm, respectively. We demonstrate that phase-locked Kerr frequency combs can be generated by pumping at any resonances in the optical C-band. The spectra spanning more than 20 THz (full width at −20 dB) are smooth without periodic amplitude modulations.

For applications such as high speed communication and astrospectrograph calibration, it is beneficial to have a broadband optical frequency comb with a smooth and flat spectral shape^32^,^33^. Such an unstructured spectrum is difficult to attain in a multi-mode microresonator (Fig. 1), especially in Si_3_N_4_ microresonators where mode coupling is facilitated by the strong sidewall scattering^26^. Reducing the waveguide cross-section to 1 × 0.8 μm^2^ is a solution to eliminate higher order modes, but it results in a larger optical mode overlap with the waveguide boundary and consequently lowers the achievable quality factor. Compared to the multi-mode microresonator (*Q*_*I*_ = 1,560,000), the microresonator made of a 1 × 0.8 μm^2^ waveguide only has an intrinsic quality factor of 830,000 (Table 1). Such decrease in the intrinsic quality factor leads to a reduction of cavity-enhanced Kerr effect by 3.5 times 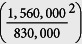
10. Furthermore, such single-mode waveguide features positive GVD and large third-order dispersion (TOD, *β*_*3*_) for all optical communication wavelengths (Fig. 2a,b), inhibiting the Kerr frequency comb and dissipative cavity soliton generation.

Our strategy to suppress higher order modes while maintaining high quality factor and anomalous dispersion is two-fold (Fig. 2c). First, a uniform single-mode waveguide (1 × 0.8 μm^2^) is included in the semi-circle region where the waveguide is curved, as bending introduces significant mode coupling if a multi-mode waveguide is used. The diameter of the semi-circle is 200 μm. Second, the microresonator is enclosed by adding straight waveguides with adiabatically tapered widths to join the two semi-circles. Each straight waveguide is 800 μm long with the width tapered linearly from 1 μm at the two ends to 2 μm at the middle, ensuring selective excitation of the fundamental mode in the otherwise multi-mode waveguide segment. The tapered waveguide accounts for >70% of the cavity length, thus the quality factor is minimally compromised (Table 1) and the average GVD remains to be anomalous in this novel Si_3_N_4_ microresonator (Fig. 2a). The average TOD is calculated as −155 fs^3^/mm (Fig. 2b) and its perturbative effect on the dissipative cavity soliton is evaluated by introducing the normalized TOD coefficient 
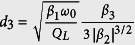
, where *β*_*1*_ is the group velocity and *Q*_*L*_ is the loaded quality factor^34^. In the designed microresonator, *d*_3_ = −0.016 ≪ 1 and thus it is still in the GVD-dominant regime that is beneficial for soliton formation and symmetric Kerr frequency comb generation. By numerically solving the Lugiato-Lefever equation (LLE) which describes the dynamics of dissipative cavity solitons^35^, it is shown that a stable 50-fs long pulse can be generated from the designed miroresonator (Fig. 2d).

The microresonator was fabricated with CMOS-compatible processes: first, a 5 μm thick under-cladding oxide was deposited to suppress substrate loss. An 800 nm thick Si_3_N_4_ layer was then deposited via low-pressure chemical vapor deposition, patterned by optimized deep-ultraviolet lithography, and etched via optimized reactive ion dry etching. Annealing at a temperature of 1150 °C was then applied to the chip for 3 hours to reduce the waveguide propagation loss. Finally the silicon nitride spiral resonators were over-cladded with a 3 μm thick oxide layer. Characterization of the fabricated microresonator is summarized in Fig. 3. A high resolution coherent swept wavelength interferometer (SWI) was implemented to characterize the cold cavity properties, *Q* and GVD, of the microresonator^17^. The wavelength of a tunable external cavity diode laser (ECDL) was linearly tuned from 1530 nm to 1590 nm at a scan speed of 60 nm/s. The laser was set to be purely TE polarized by inserting a polarizer with an extinction ratio of >100 before the coupling optics. Similarly, an analyzer was used at the output to guarantee exclusive collection of either TE polarized (Fig. 3a) or TM polarized (Fig. 3c) transmission spectra. To ensure uniform optical frequency sampling of 7 MHz, 10% of the ECDL output was tapped to an unbalanced Mach-Zehnder interferometer, and the photodetector output was used as the sampling clock of the data acquisition. To minimize the thermally induced resonance shift, the on-chip temperature was actively controlled to ensure the temperature drift in 1 second was within 1 mK. The accuracy of our ECDL is 200 MHz, and thus an absolute optical frequency calibration is necessary for precise characterization of the cold cavity properties. 1% of the ECDL output was thus tapped to a fiber coupled hydrogen cyanide (HCN) gas cell and the transmission spectrum of the gas cell was recorded simultaneously with that of the microresonator. Figure 3a shows the cold cavity transmission of the microresonator, calibrated with 51 absorption features of the HCN gas cell. No higher order transverse modes are observed throughout the optical C/L bands in the spectrum. The inset shows the resonance at 1556 nm, which is undercoupled with a loaded *Q* of 1,000,000 and intrinsic *Q* of 1,360,000. 10 transmission spectra were taken and independently fitted to determine the mean value of the GVD and the error bar of the dispersion measurements. Figure 3b shows the wavelength dependence of the free spectral range (FSR), measuring a FSR of 64.24 GHz and a mode non-equidistance of 196 ± 10 kHz at 1556 nm. The GVD extracted from the measurements is anomalous at −50 ± 2.6 fs^2^/mm.

A closer look at the transmission spectrum reveals that the two resonances around 1558 nm are hybridized modes from polarization coupling (green lines in Fig. 3a). Such polarization coupling mainly originates from fabrication imperfections such as surface roughness and sidewall angle^30^. The strength of the polarization coupling is rather weak and the slight dispersion disruption around 1558 nm is negligible in the Kerr frequency comb formation, as evidenced by the smooth spectral shapes shown in Fig. 4. Moreover, the 7.5 THz period of the polarization coupling is comparably larger than the 1.5 THz cycle of the higher order mode coupling. Thus the polarization coupling occurs less frequently and introduces fewer perturbations to dissipative soliton formation. Since coupling strength is inversely proportional to the difference in resonance frequencies of the interacting TE and TM modes, we further identify the on-chip temperature as a convenient control parameter to adjust the impact of polarization coupling. For instance, the cavity loading can be changed from 65% (right inset of Fig. 3a) to 40% (Fig. 3c) via increasing the on-chip temperature by 7 °C with a thermoelectric cooling device.

For Kerr frequency comb generation, the microresonator was pumped with an on-chip power up to 1.7 watts. A flat and smooth phase-locked Kerr frequency comb can be generated by pumping at any resonances in the optical C-band, thanks to the absence of quality factor and dispersion disturbance induced by mode couplings. Figure 4a shows three example spectra pumping at 1551.83 nm, 1556.48 nm, and 1561.58 nm. Importantly, the spectral shapes resemble one another and periodic amplitude modulations are not observed, distinctly different from Fig. 1 where the comb spectrum is structured by the mode coupling characteristics. Tuning speed of the pump frequency has been shown to be a critical parameter to drive the Kerr frequency comb into low-phase noise soliton state, circumventing the thermal effect of the microresonator^16^. To find the proper tuning speed, pump power transmission was monitored while pump frequency was scanned, via control of the piezoelectric transducer, across a cavity resonance with tuning speeds varying from 1 nm/s to 100 nm/s. When the tuning speed was higher than 40 nm/s, a characteristic step signature of low-phase noise soliton state was observed. Figure 4b shows the pump power transmission with the pump frequency tuning speed set at 60 nm/s. Coherence of the Kerr frequency comb was characterized by measuring the RF amplitude noise with a scan range much larger than the cavity linewidth^15^,^36^,^37^. Pump mode was removed with a fiber WDM filter to avoid saturation of the photodetector. Figure 4c shows RF amplitude noise of the Kerr frequency comb at different states, showing the transition from a high-phase noise state (state 1) to the low-phase noise soliton state (state 2). In state 3, the pump was off-resonance and Kerr frequency comb was not generated. At the proper pump-resonance detuning, the RF amplitude noise dropped by more than two orders of magnitude (state 1 to state 2) and reached the background noise of the photodetector (state 3). The RF amplitude noise measurements confirm the generation of phase-locked Kerr frequency combs.

In summary, we report a novel design of Si_3_N_4_ microresonator which can be used to attain single-mode operation, high quality factor, and anomalous dispersion all simultaneously. The microresonator is consisted of uniform single-mode waveguides in the semi-circle region, to eliminate bending induced mode coupling, and adiabatically tapered waveguides in the straight region, to ensure selective excitation of the fundamental mode. The intrinsic *Q* of the novel single-mode microresonator is 1.36 × 10^6^, 1.6 times larger than that of a conventional single-mode microresonator. More importantly, the GVD remains to be anomalous at −50 fs^2^/mm. With the novel single-mode microresonator, we demonstrate the generation of phase-locked Kerr frequency combs pumped at any resonances in the optical C-band. The spectra spanning more than 20 THz (full width at −20 dB) are smooth and flat without periodic amplitude modulations.

## Additional Information

**How to cite this article**: Huang, S.-W. *et al*. Smooth and flat phase-locked Kerr frequency comb generation by higher order mode suppression. *Sci. Rep.* **6**, 26255; doi: 10.1038/srep26255 (2016).

## Figures and Tables

**Figure 1:**
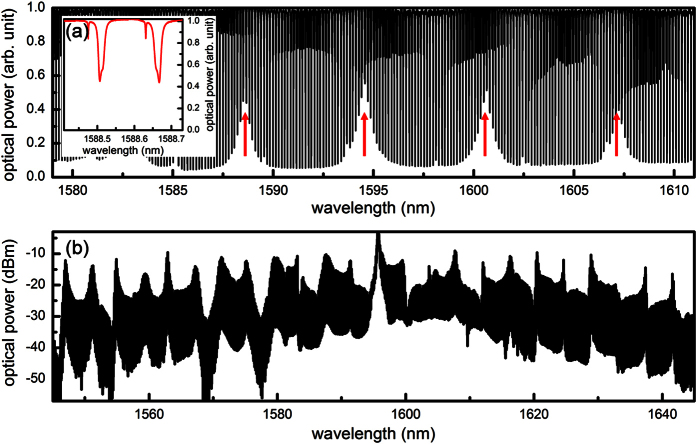
Kerr frequency comb from a multi-mode microresonator. (**a**) Cold cavity transmission of a multi-mode microresonator. The waveguide cross-section is uniform at 2 × 0.8 μm^2^ along the whole microresonator. Five modal families (3 TE and 2 TM) are identified and mode hybridization between the first two TE modes leads to periodic disruptions in dispersion and quality factor (red arrows). Inset: Zoom-in view of the cavity resonances around 1588.6 nm. (**b**) Example Kerr frequency comb spectrum, showing a periodic amplitude modulation in its spectral shape due to the mode hybridization.

**Figure 2:**
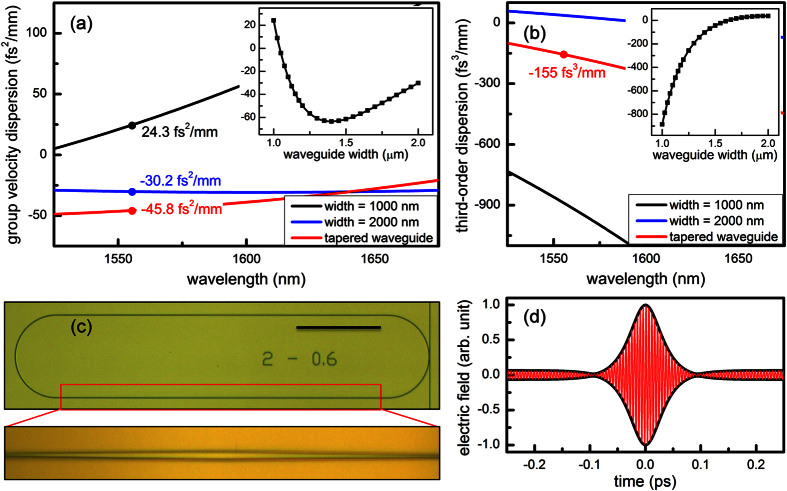
Design of the novel single-mode microresonator. (**a,b**) Group velocity dispersion and third-order dispersion of the uniform waveguides with different widths and the tapered waveguide calculated with a commercial full-vector finite-element-mode solver (COMSOL Multiphysic), taking into consideration both the waveguide dimensions and the material dispersion^38^. To account for the tapered geometry, waveguide dispersions are calculated for various widths (1 to 1.2 μm with a step of 25 nm and 1.25 to 2 μm with a step of 50 nm) and dispersions as a function of waveguide width is obtained using a cubic spline interpolation (insets). The fitted functions are then used to calculate the path-averaged dispersions 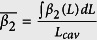 and 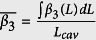, where *dL* = 400*nm*. (**c**) An optical micrograph of the designed single-mode microresonator. The waveguide in the semi-circle regions has a uniform width of 1 μm, supporting only the fundamental modes. On the other hand, the 800 μm long straight waveguide has a tapered width from 1 μm at the end to 2 μm at the middle of the waveguide. The total cavity length is 2.2 mm. Scale bar: 200 μm. (**d**) Dissipative cavity soliton with a FWHM duration of 50 fs is obtained by numerically solving the Lugiato-Lefever equation (LLE), 
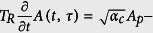

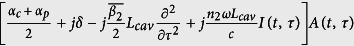
, where *A*(*t, τ*) is the envelope function of the dissipative cavity soliton, *α*_*p*_ is the propagation loss, *α*_*c*_ is the coupling loss, *δ* is the pump-resonance detuning, and *T*_*R*_ is the cavity roundtrip time. 2,001 modes centered at the pump are incorporated in the LLE modeling. The simulation starts from vacuum noise and is run for 1.5 × 10^5^ roundtrips until the solution reaches the steady state.

**Figure 3:**
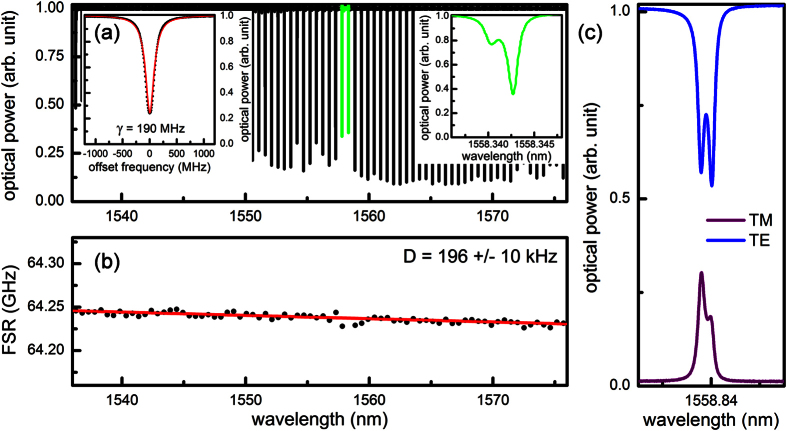
Characterization of the novel single-mode microresonator. (**a**) Cold cavity transmission of the designed single-mode microresonator, measured with the high resolution coherent swept wavelength interferometer. Higher order modes are not observed in the microresonator, but the weak TE and TM coupling around 1558 nm results in a 10% reduction in the cavity loading (green lines and inset on the right). Inset on the left: resonance at 1556 nm is undercoupled with a loaded *Q* of 1,000,000. (**b**) Wavelength dependence of the free spectral range (FSR), measuring a non-equidistance of the modes, 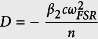, of 196 ± 10 kHz. The extracted group velocity dispersion is anomalous at −50 ± 2.6 fs^2^/mm. The slight dispersion disruption around 1558 nm is negligible in the Kerr frequency comb formation, evidenced by the smooth spectral shapes shown in Fig. 4. (**c**) At a different temperature (ΔT = 7 °C) where the TE and the TM resonances become closer to degeneracy, stronger polarization coupling results in further reduction in the cavity loading. The input is TE polarized and the output is analyzed by either a TE or a TM polarizer, showing the hybridized modes are superposition of both polarization states.

**Figure 4:**
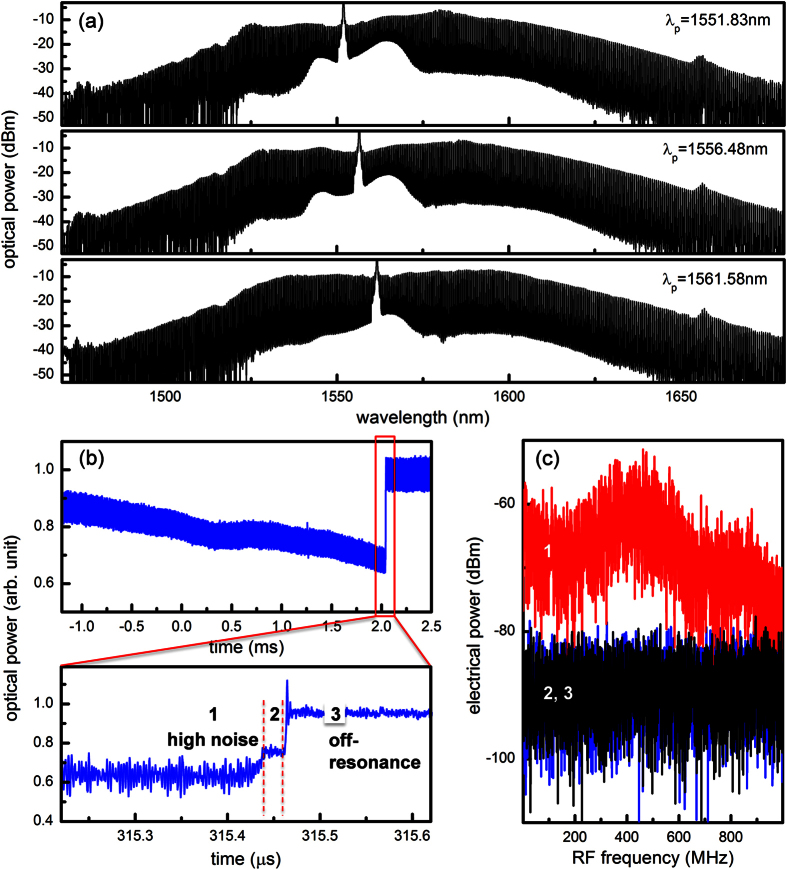
Smooth phase-locked Kerr frequency combs pumped at different wavelengths. (**a**) In the designed single-mode microresonator, phase-locked Kerr frequency combs can be generated by pumping at any resonances in the C-band. Three example spectra are shown here. Moreover, the spectral shapes are smooth without periodic amplitude modulations, distinctly different from Fig. 1. (**b**) Pump power transmission as the pump wavelength is scanned across a cavity resonance at a speed of 60 nm/s. The observation of the step signature is characteristic of the low-phase noise soliton state (state 2). (**c**) RF amplitude noise of the Kerr frequency comb at different states (1: high-phase noise, 2: low-phase noise) along with the detector background (3), showing the transition in and out of the low-phase noise state. The scan range of 1 GHz is more than five times the cavity linewidth.

**Table 1:** Intrinsic and loaded quality factors of different types of microresonators.

Microresonator type	loaded *Q*	intrinsic *Q*
Single-mode waveguide with a uniform width of 1 μm	0.6 M	0.83 M
Multi-mode waveguide with a uniform width of 2 μm	1.1 M	1.56 M
Single-mode waveguide with varying widths from 1 to 2 μm	1.0 M	1.36 M

For a fair comparison, all other parameters of the microresonators are kept the same.
